# Supplementary material for: A comprehensive map of the aging blood methylome in humans
Source: Genome Biol. 2024 Sep 6;25:240. doi: 10.1186/s13059-024-03381-w (PMC11378482; doi:10.1186/s13059-024-03381-w)
Supplement: Supplementary file 1 — Additional file 1: The supplementary figures for this publication. [file 13059_2024_3381_MOESM1_ESM.pdf]

A comprehensive map of the ageing blood methylome in humans

Kirsten Seale[1], Andrew Teschendorff[2,3], Alexander P Reiner[4], Sarah Voisin[1,5]* and Nir Eynon[5*]

[1]Institute for Health and Sport (iHeS), Victoria University, Footscray, VIC 3011, Australia.
[2]CAS Key Lab of Computational Biology, Shanghai Institute of Nutrition and Health, Chinese Academy of Sciences, Shanghai 200031, China
[3]UCL Cancer Institute, University College London, London, United Kingdom
[4]Department of Epidemiology, University of Washington, Seattle, WA, USA.
[5]Australian Regenerative Medicine Institute, Monash University, Clayton, VIC, Australia

Supplementary figures

Fig S1. Sample size and age distribution in the 56 blood datasets analysed in this study.

Fig S2. Doughnut chart of the proportions of highly, intermediately, and lowly methylated CpGs with age.

Fig S3. Enrichment of hypomethylated and hypermethylated differentially methylated positions (DMPs) and non-DMPs in chromatin states, biological pathways and epigenetic clocks.

Fig S4. Comparison of DMP and VMP meta-analyses with and without correction for cell types.

Fig S5. Enrichment of three classes of age-associated CpGs in chromatin states, biological pathways and epigenetic clocks.

Fig S2. Forest plot of the genome-wide meta-analysis of entropy and age in blood.

Fig S7. A comparison of the changes in entropy with age in homoscedastic DMPs, DMPs-VMPs and constant VMPs

Fig S8. Entropy changes in blood after correction for cell types.

Fig S9. Entropy comparison in six sorted blood cell types in a single dataset.

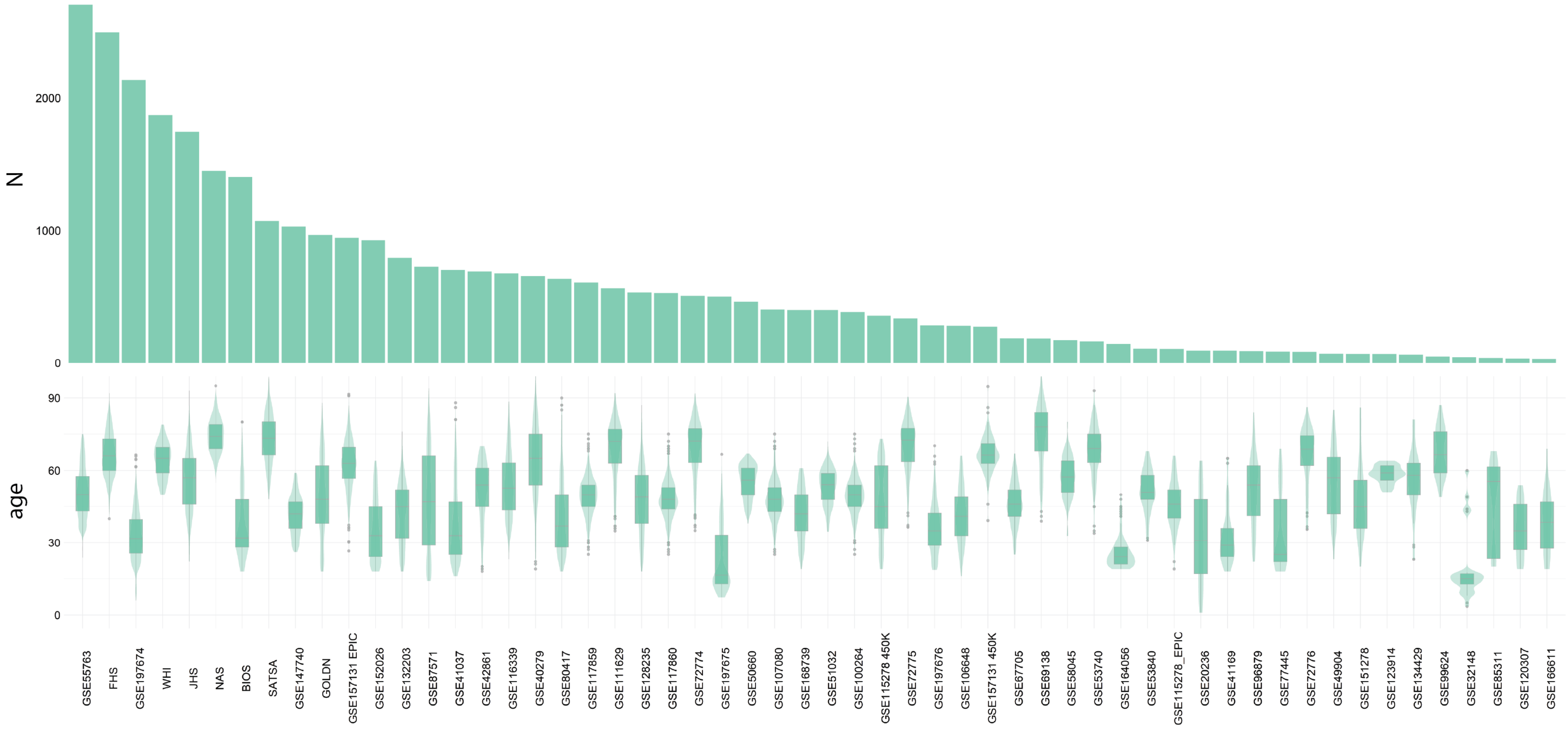

Fig S1. Sample size and age distribution in the 56 blood datasets analysed in this study. Bottom panel: boxplots & violin plots displaying the distribution of samples across the age range. Top panel: number of samples in each dataset (N), ordered from largest (left) to smallest (right).

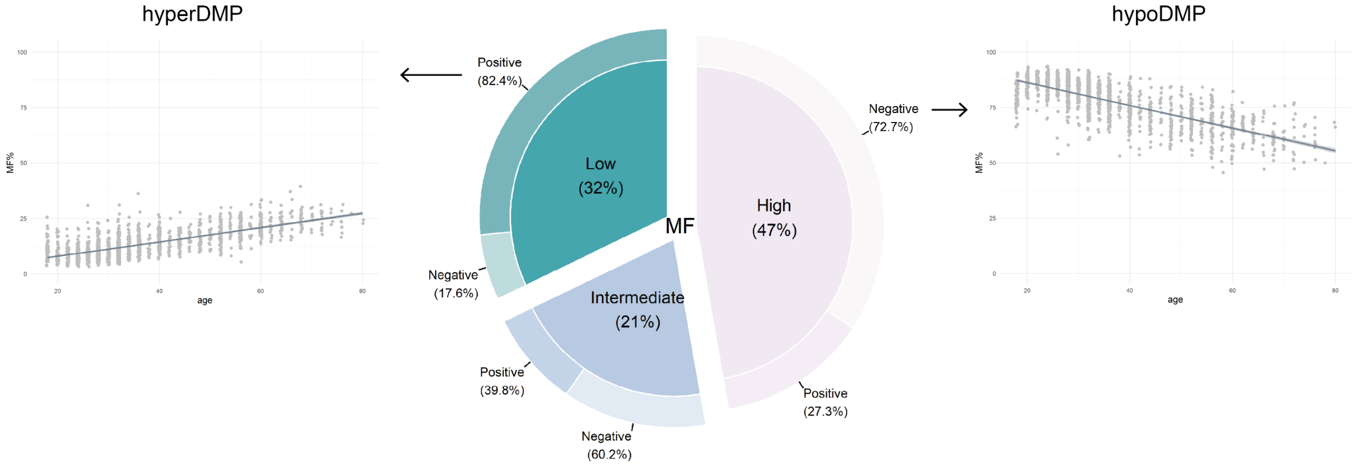

Fig S2. Doughnut chart of the proportions of highly, intermediately, and lowly methylated CpGs with age. The inner pie of the doughnut represents the proportions of CpGs that are highly methylated (<75%), intermediately methylated (25 – 75%) and lowly methylated (<25%) at baseline in a single blood dataset (BIOS), with the direction of change in methylation with age in the outer circle. A positive direction implies that a DMP increases in methylation with age, and a negative direction implies a DMP loses methylation with age. MF = Methylation Fraction

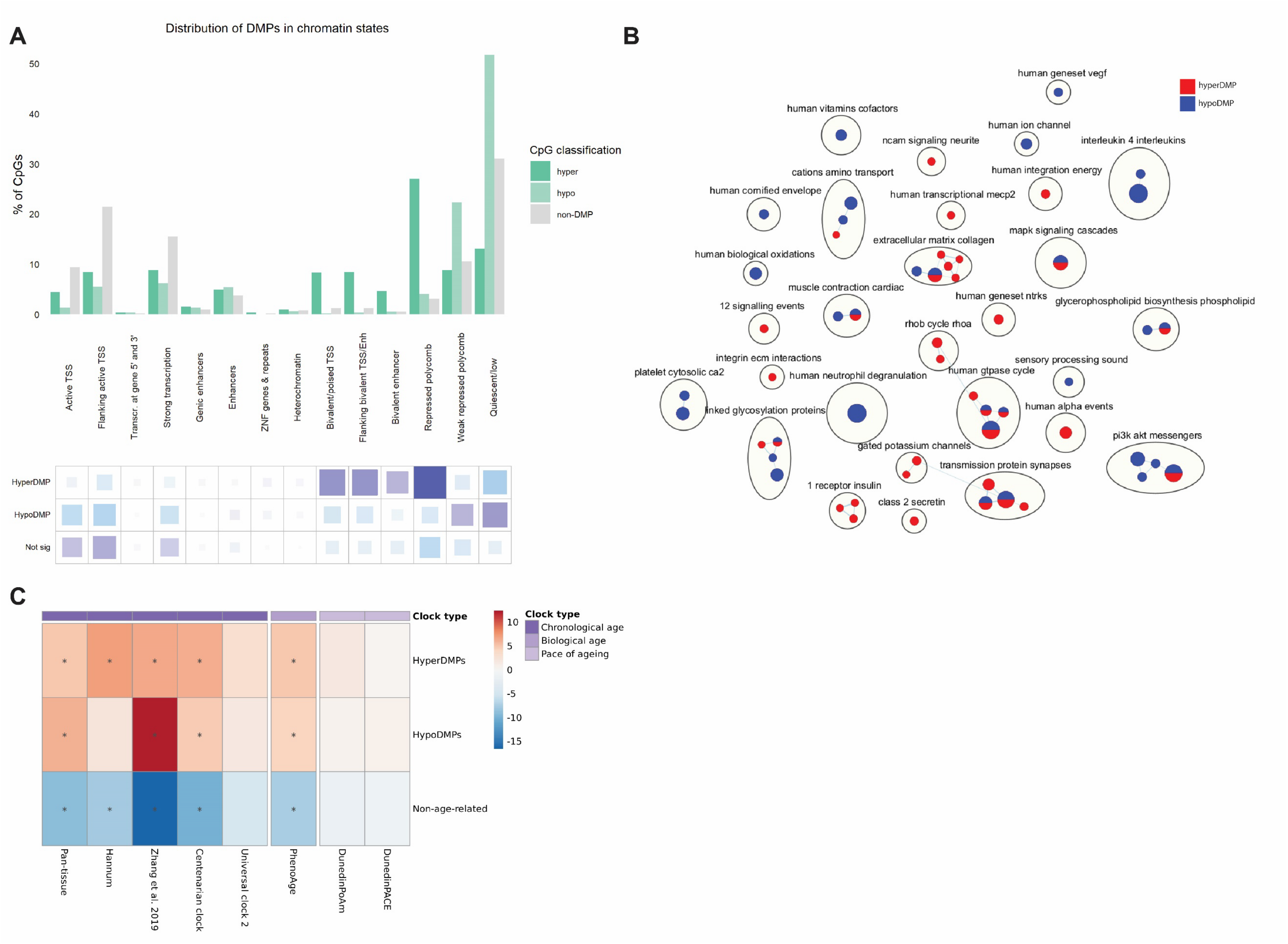

Fig S3. Enrichment of hypomethylated and hypermethylated differentially methylated positions (DMPs) and non-DMPs in chromatin states, biological pathways and epigenetic clocks. A) Distribution of hypoDMPs, hyperDMPs and non-DMPs in chromatin states from peripheral blood mononuclear cells (PBMCs); the grids under the graph represent the residuals from the χ^2 test, with the size of the blocks in the grid being proportional to the cell's contribution. Purple indicates over-representation or enrichment in the chromatin state, and blue represents under-representation or depletion in the chromatin state. **B)** Enrichment map showing Reactome pathways enriched in hypomethylated or hypermethylated DMPs. Nodes in the network represent pathways and similar pathways with many common genes are connected. Groups of similar pathways are indicated. Nodes are coloured according to significance in different DMPs types: blue (hypomethylated) and red (hypermethylated). **C)** Relative over- (red) or under- (blue) representation of clock CpGs in hyperDMPs, hypoDMPs and non-age-related CpGs.

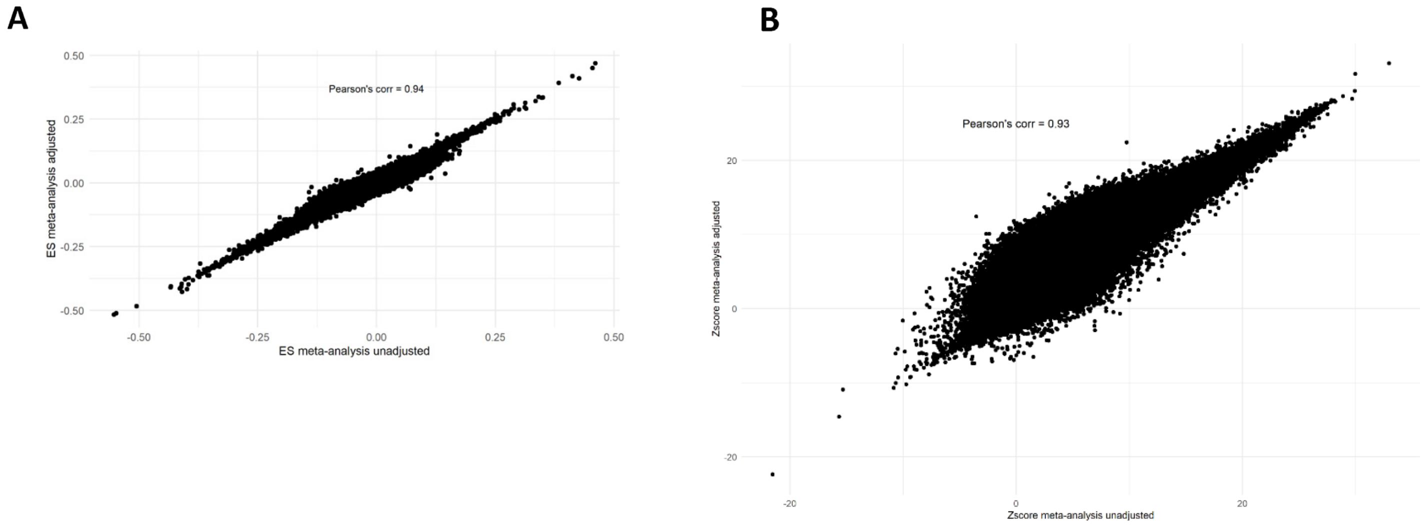

Fig S4. Comparison of DMP and VMP meta-analyses with and without correction for cell types. A) A correlation plot of the effect sizes (ES) of the CpGs meta-analysed in the cell type adjusted versus the meta-analysis not adjusted for cell types of differential methylation and age in blood. The ES from the meta-analysis not adjusted for cell types on the x-axis and the ES for the meta-analysis adjusted for cell types on the y-axis. **B)** A correlation plot of the Zscores of the CpGs in the variable methylation and age meta-analyses in both the cell type adjusted and the unadjusted meta-analysis in blood. The Zscore from the meta-analysis not unadjusted for cell types on the x-axis and the Zscore for the meta-analysis adjusted for cell types on the y-axis.

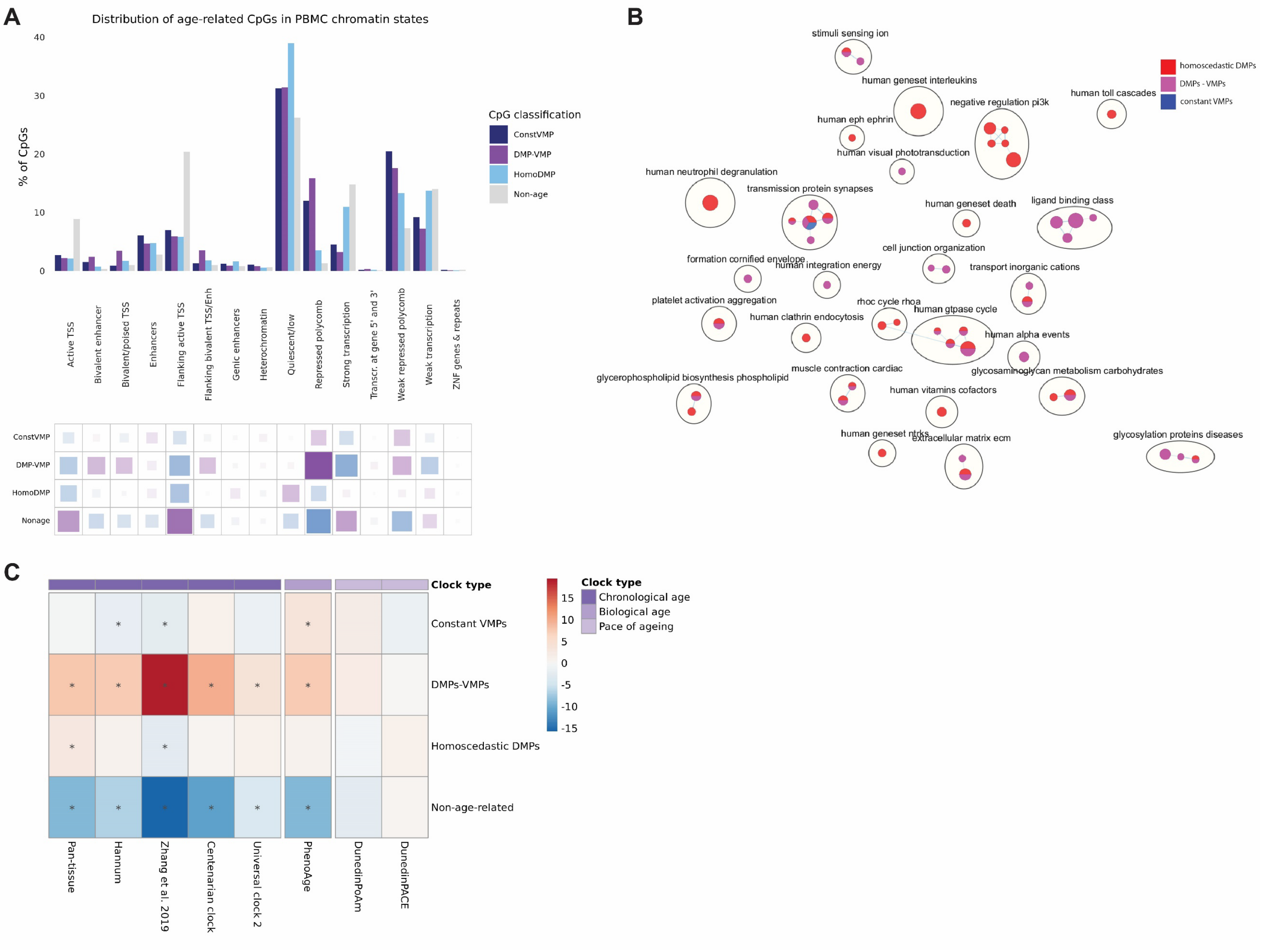

Fig S5. Enrichment of three classes of age-associated CpGs in chromatin states, biological pathways and epigenetic clocks. A) Distribution of homoscedastic DMPs, DMPs-VMPs, constant VMPs and non-age-related CpGs in chromatin states from PBMCs. The grids under the graph represent the residuals from the χ^2 tests, with the size of the blocks in the grid being proportional to the cell's contribution. Purple indicates over-representation or enrichment in the chromatin state, and blue represents under-representation or depletion in the chromatin state. **B)** Enrichment map showing Reactome pathways enriched in homoscedastic DMPs, DMPs-VMPs, or constant VMPs. Nodes in the network represent pathways and similar pathways with many common genes are connected. Groups of similar pathways are indicated. Nodes are coloured according to significance in different age-related CpG types: red (homoscedastic DMPs), purple (DMPs-VMPs) and blue (constant VMPs). **C)** Relative over- (red) or under- (blue) representation of clock CpGs in homoscedastic DMPs, DMPs-VMPs, constant VMPs and non-age-related CpGs.

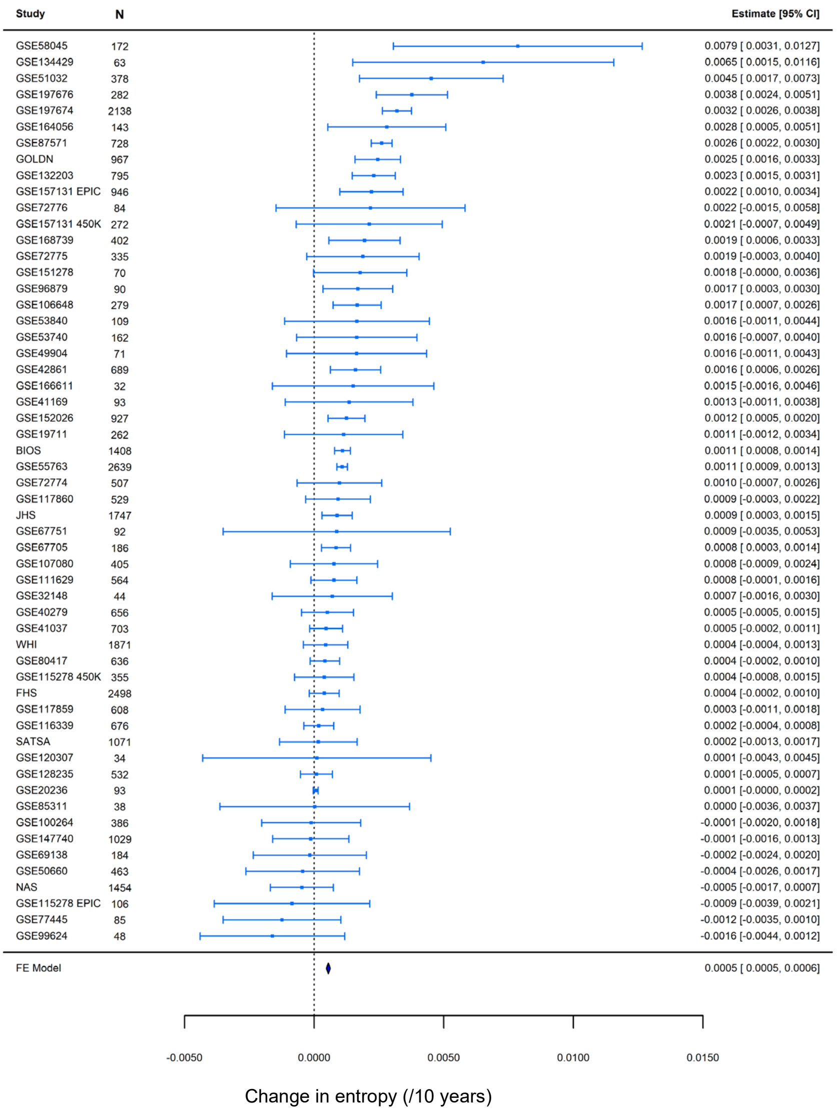

Fig S6. Forest plot of the genome-wide meta-analysis of entropy and age in blood. On the x-axis is the change in entropy per decade of age, with the effect size and standard errors from each independent epigenome-wide association study (EWAS) plotted on the y-axis. The dataset, sample size (N) and mean age ± SD (standard deviation) are on the left side of the plot and the 95% confidence intervals (CI) are displayed on the right panel. The meta-analysis effect size is represented by the blue polygon.

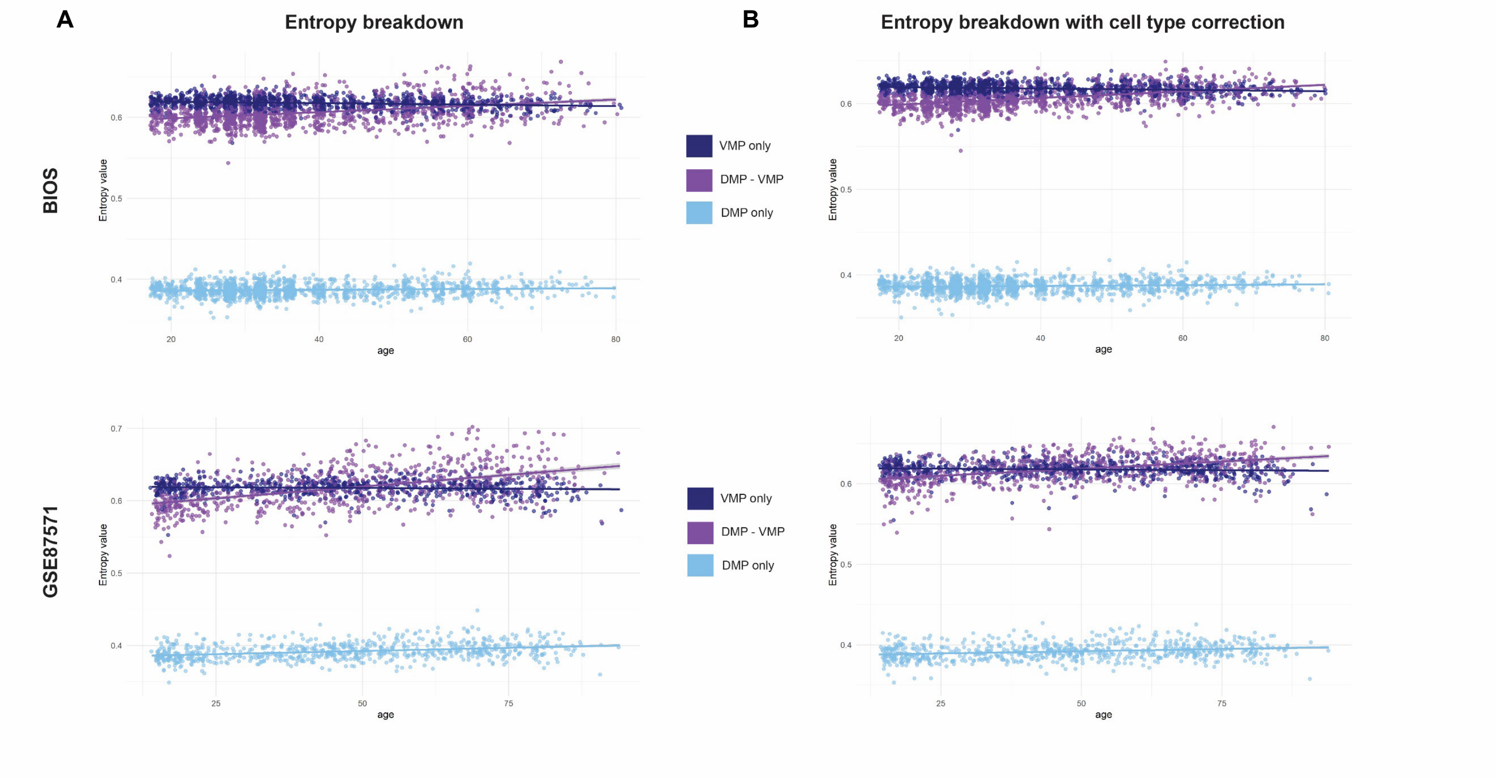

Fig S7. A comparison of the changes in entropy with age in homoscedastic DMPs, DMPs-VMPs and constant VMPs. Entropy was calculated for each type of age-related CpG on the y-axis and plotted against age (x-axis) for two datasets: BIOS and GSE87571. Analyses were performed A) unadjusted for cell types and B) adjusting for cell types.

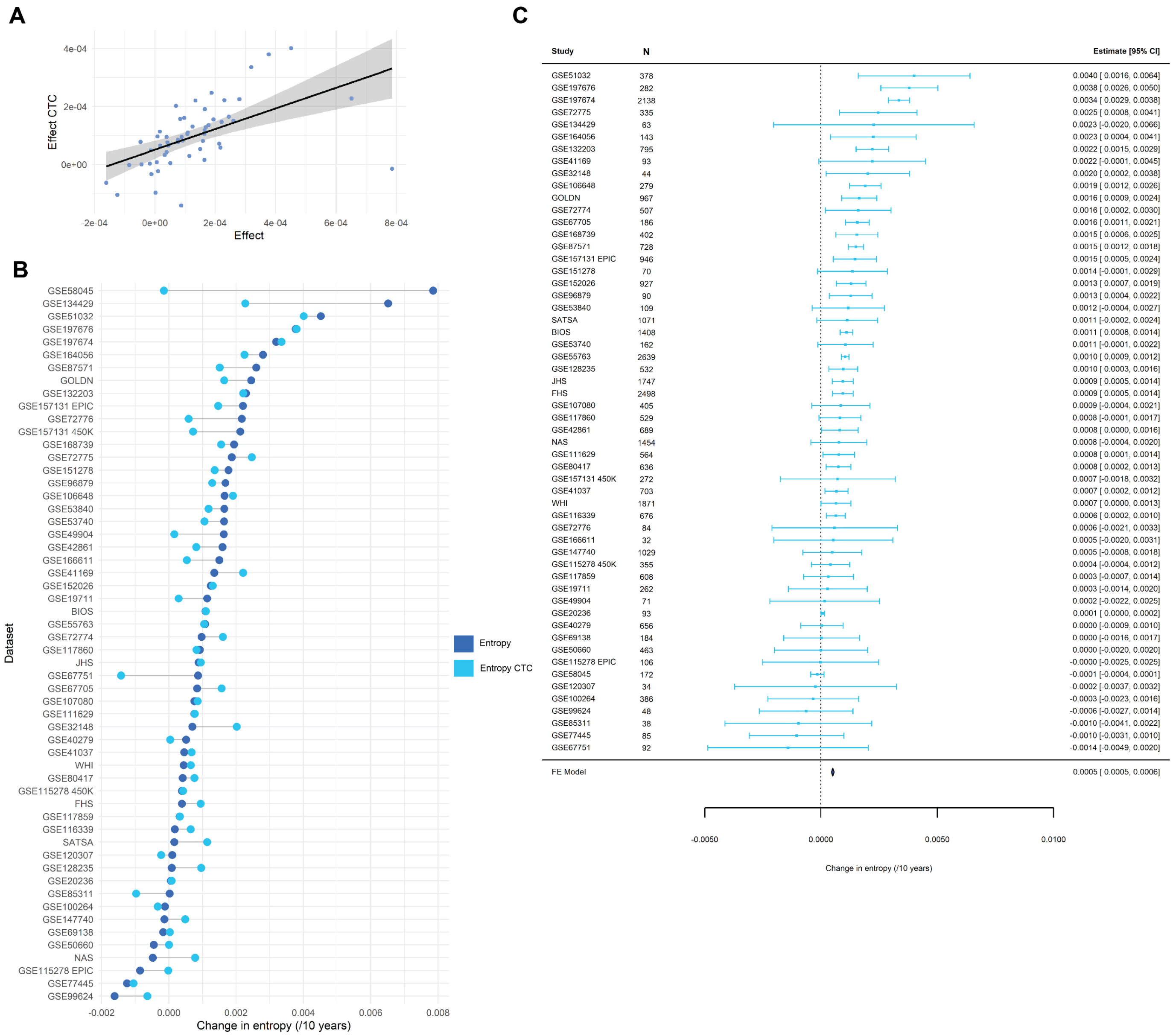

Fig S8. Entropy changes in blood after correction for cell types. A) The effect sizes from the independent epigenome-wide association study (EWAS) of entropy and age for the analyses unadjusted for the 5 largest blood cell types (x-axis) and the effect size for the cell type corrected (CTC) analyses. Each point on the graph represents a single blood dataset. The Pearson's correlation is 0.53. **B)** A lollipop chart to display the change in direction of entropy before (royal blue) and after cell type correction (skyblue) for each dataset (y-axis). **C)** A forest plot of the genome-wide meta-analysis of entropy and age in the cell type corrected datasets. The change in entropy per decade of age is located on the x-axis, with the effect size and standard error measurements from the independent EWAS on the y-axis. The 95% confidence intervals (CI) are on the right pane. The meta-analysis effect size is represented by the navy polygon at the bottom of the plot.

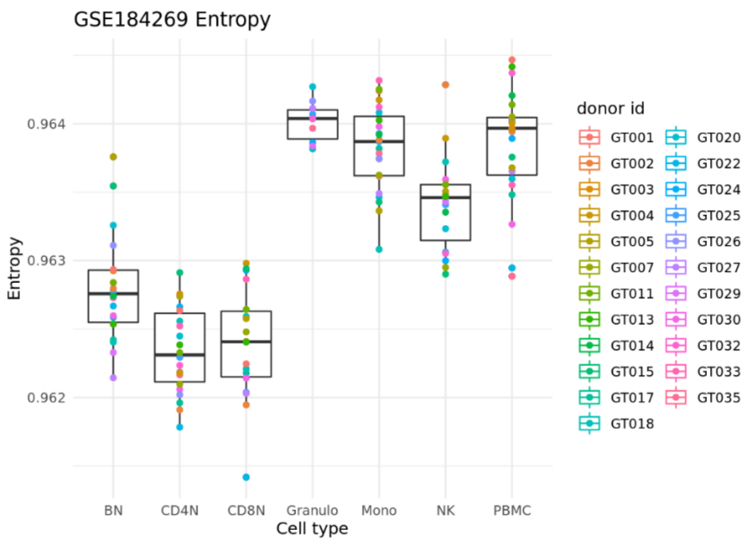

Fig S9. Entropy comparison in six sorted blood cell types in a single dataset. Individual entropy values (y-axis) for six sorted cell types (naïve B cells, naïve CD4+ T cells, naïve CD8+ T cells, granulocytes, monocytes, natural killer cells) and peripheral blood mononuclear cells (PMBCs) (x-axis) from the GSE184269 dataset that contains both mixed and sorted blood cells from the same individuals. Each individual is colour-coded according to the legend (donor id) on the right side of the chart.
