# Supplementary material for: A comprehensive map of the aging blood methylome in humans
Source: Genome Biol. 2024 Sep 6;25:240. doi: 10.1186/s13059-024-03381-w (PMC11378482; doi:10.1186/s13059-024-03381-w)
Supplement: Supplementary file 2 — Additional file 2: The supplementary tables for this publication. [file 13059_2024_3381_MOESM2_ESM.pdf]

A comprehensive map of the ageing blood methylome in humans

Kirsten Seale[1], Andrew Teschendorff[2,3], Alexander P Reiner[4], Sarah Voisin[1,5]* and Nir Eynon[5]*

[1]Institute for Health and Sport (iHeS), Victoria University, Footscray, VIC 3011, Australia.
[2]CAS Key Lab of Computational Biology, Shanghai Institute of Nutrition and Health, Chinese Academy of Sciences, Shanghai 200031, China
[3]UCL Cancer Institute, University College London, London, United Kingdom
[4]Department of Epidemiology, University of Washington, Seattle, WA, USA.
[5]Australian Regenerative Medicine Institute, Monash University, Clayton, VIC, Australia

Supplementary tables

Table S1. Description of blood datasets.

Table S2. Shannon entropy results in blood.

Table S3. Entropy results for three categories of age-associated CpGs.

Table S4. Entropy results in datasets containing isolated cell types.

Table S1. Description of blood datasets.

Dataset name	Database	Reference	Initial N	Sample size after pre-processing	Age (mean ± SD)	Age range (min - max)	Array	% Male	Phenotype	Access / info required	Check for sex	Pre-processed ourselves	Covariates
GOLDN	dbGaP	10	967	967	49 ± 16	18 - 88	450K	47.4	Normal	Yes. Access through dbGaP.	Yes	Yes	age + sex
WHI	dbGaP	11	1890	1871	64 ± 7.0	50 - 79	450K	0.0	Postmenopausal women	Yes. Access through dbGaP.	N/A. All samples are female.	Yes	age
GSE55763	GEO	12	2707	2639	51 ± 10	23 - 75	450K	67.7	Normal		Yes. 4 samples with incorrect sex removed.	No. R keeps crashing when trying to pre-process.	age + sex
GSE128235	GEO	13	537	532	48 ± 13	18-87	450K	42.7	Depression / healthy control		Yes.	Yes. 1 sample corrupted IDAT file. 4 samples without a diagnosis removed.	age + sex + diagnosis
GSE99624	GEO	14	48	48	67 ± 9.9	49 - 87	450K	18.7	Osteoporosis / healthy controls		Yes	Yes	age + sex
GSE115278_450	GEO	15	366	355	47 ± 15	19 - 73	450K	35.8	Normal		Yes. 11 samples removed with incorrect sex.	Yes	age + sex + study
GSE115278_EPIC	GEO	15	108	106	46 ± 9.6	19 - 66	EPIC	36.8	Normal		Yes. 2 samples removed with incorrect sexes	Yes	age + sex + study
GSE87571	GEO	16	732	728	47 ± 21	14 - 94	450K	46.7	Normal		Yes. 3 samples removed with incorrect sex.	Yes. 1 sample missing age information.	age + sex
GSE53740	GEO	17	384	162	68 ± 10	34 - 93	450K	48.4	Progressive supranuclear palsy / frontotemporal dementia / healthy controls		Yes. Remove batch 2 all samples have been scrambled. Remove 63 samples from batch 1 with mismatch sex or undefined sex.	Yes	age + sex + race
GSE58045	GEO	18	172	172	57 ± 8.2	32 - 80	27K	0.0	Normal twins		N/A. All samples female.	No	age + (1|family number)
NAS	dbGaP	19	1454	1454	75 ± 7.0	55 - 100	450K	100.0	Normal / chronic disease	Yes. Access through dbGaP.	N/A. All samples are male.	Partly. Raw files not available but ran normalisation and batch correction.	age + year + (1|ID)
BIOS	EGA	20	4386	1408	38 ± 14	18 - 80	450K	34.2	Unknown	Yes. Application sent to authors.	Not all sex info supplied. 3 samples with sex did not match predicted sex.	Yes. Samples without ages remove, samples with corrupt IDATs removed.	age + sex
GSE77445	GEO	21	85	85	34 ± 16	18 - 69	450K	50.5	Normal		No. Only signal intensities for a reduced set of CpGs are provided.	No	age + sex
JHS	Independent	22	1747	1747	56 ± 12	22 - 93	EPIC	37.0	Cardiometabolic conditions in African population	Yes. Independent application.	Yes.	No	age + sex + education + BMI + smoker + alcohol
FHS	dbGaP	23	2562	2498	66 ± 8.9	40 - 92	450K	45.8	Normal / cardiovascular disease	Yes. Access through dbGaP.	Yes. 19 samples removed	Yes. 40 Samples had corrupt IDATS removed. 1 sample failed QC.	age + sex
GSE49904	GEO	24	71	71	55 ± 15	23 - 83	27K	31.0	Iron deficiency anaemia / Anaemia / Healthy		N/A	No	age + sex + ethnicity + diagnosis + smoking history
GSE80417	GEO	25	675	636	40 ± 15	18 - 90	450K	59.3	Schizophrenia / normal		Yes.	No. 39 samples with no age / incorrect age.	age + sex
SATSA	ArrayExpress	26	1072	1071	73 ± 9.7	48 - 98	450K	39.7	Normal		Yes. 1 sample removed	Yes	age + sex + (1|twin pair)
GSE42861	GEO	27	689	689	52 ± 12	18 - 70	450K	28.6	Rheumatoid arthritis / healthy controls		Yes	Yes	age + sex + disease status
GSE51032	GEO	28	845	378	53 ± 7.2	34-72	450K	20.4	Only normal samples		Yes. 10 samples incorrect sex removed.	No. IDATS corrupted. Removed 424 samples with a cancer diagnosis. 9 samples failed QC	age + sex
GSE67705	GEO	29	284	186	46 ± 8.8	25 - 67	450K	100.0	HIV positive / HIV negative		N/A. All samples male.	No. 98 Samples failed QC and were removed.	age + HIV status
GSE32148	GEO	30	48	44	20 ± 15	10-76	450K	47.9	Normal / Crohn's / Ulcerative Colitis		Yes. 4 samples removed with incorrect sex.	No	age + sex + disease status
GSE106648	GEO	31	279	279	41 ± 11	16 - 66	450K	27.6	Multiple sclerosis / healthy control		Yes	Party. No detection pvalue information.	age + sex + smoking status
GSE69138	GEO	32	589	184	75 ± 13	39 - 99	450K	51.1	Ischaemic stroke subtypes		Yes. 1 sample removed with incorrect sex	Yes	age + sex + stroke subtype
GSE50660	GEO	33	464	463	55 ± 6.6	38 - 67	450K	70.5	Smoker / non-smoker		Yes. 1 sample removed.	No. Sample IDs in raw files don't match the phenotype table.	age + sex + smoking status
GSE40279	GEO	34	656	656	64 ± 15	19 - 101	450K	48.5	Healthy	Emailed authors for raw IDATS or detection pvals.	No	No. Could not pre-process. Raw sample IDs did not match the phenotype table.	age + sex + ethnicity
GSE41037	GEO	35	717	703	37 ± 16	16 - 88	27K	61.9	Schizophrenia / healthy control		Yes. 5 samples don't match	Yes. 12 samples failed QC	age + sex
GSE41169	GEO	36	95	93	32 ± 10	18 - 65	450K	29.5	Schizophrenia / healthy control	Emailed authors for raw data	Yes. 2 samples removed with incorrect sexes	No	age + sex
GSE53840	GEO	37	120	109	52 ± 8.0	31 - 68	450K	100.0	HIV viral load		Yes	Yes. 9 samples no age info.	age + HIV viral load
GSE67751	GEO	38	92	92	50 ± 8.9	24 - 68	450K	45.7	HIV / control	Emailed authors for batch info	Yes	No	age + sex + HIV status
GSE72775	GEO	39	335	335	70 ± 10	36.5-90.5	450K	58.8	Caucasian/Hispanic	Emailed authors for batch info	Yes	Partly. No batch info.	age + sex + ethnicity
GSE111629	GEO	40	572	564	69 ± 11	35 - 92	450K	56.2	Parkinson's disease / control		Yes	Yes. 8 samples failed QC.	age + sex
GSE72774	GEO	41	508	507	70 ± 11	35.1 - 92	450K	55.4	Parkinson's disease	Emailed authors for batch info	Yes. 1 sample removed with incorrect sex prediction.	Partly. No batch info.	age + sex + number of years in school + smoking status
GSE72776	GEO	42	84	84	66 ± 12	34 - 50	450K	59.5	Parkinson's disease	Emailed authors for batch info	Yes	Partly. No batch info.	age + sex
GSE166611	GEO	43	32	32	39 ± 12	19 - 69	450K	0.0	Normal weight / Obese		Yes	Yes	age + BMI
GSE164056	GEO	44	143	143	26 ± 6.4	19 - 50	EPIC	34.3	Social Anxiety Disorder		Yes	Yes	age + sex

GSE85311	GEO	45	38	38	47 ± 18	20 - 68	450K	71.0	Young / old sedentary /old exercise trained		Yes	Partly. No batch info.	age + sex + training status
GSE151278	GEO	46	70	70	47 ± 15	20-86	450K	61.4	Psoriasis		Yes	Yes	age + sex + drug response
GSE96879	GEO	47	90	90	52 ± 14	22 - 84	450K	0.0	Lupus / Healthy controls		N/A	Yes	age + ethnicity + disease status
GSE134429	GEO	48	64	63	56 ± 13	23 - 81	EPIC	11.1	Rheumatoid arthritis / healthy controls	Emailed authors for batch info	Yes. 1 sample with incorrect sex	Partly. No batch info.	age + sex + batch + patient cohort + donor
GSE120307	GEO	49	34	34	36 ± 11	19-54	450K	53.0	Healthy / psychiatric disorder (twin pairs)		Yes	No	age + sex + (1|twin pair)
GSE20236	GEO	50	93	93	32 ± 19	49 - 74	27K	0.0	Normal	Emailed authors for batch info	N/A	No. Sample IDs in the raw files are missing.	age
GSE19711	GEO	51	540	262	65 ± 6.7	52 - 78	27K	0.0	Normal samples only		N/A	Yes. Removed 266 cancer samples. 2 samples failed QC	age + ca125
GSE157131_EPIC	GEO	52	946	946	63 ± 9.7	26.4 - 91.6	EPIC	100.0	Hypertension / control	Emailed authors for batch info	All samples are male in description of study, and the sex prediction confirmed this. In the raw phenotype table, some of the samples are 'female'. Phenotypes were changed to be all male.	Partly. No batch info	age
GSE157131_450K	GEO	52	272	272	67 ± 6.5	39 - 94	450K	100.0	Hypertension / control	Emailed authors for batch info	All samples are male in description of study, and the sex prediction confirmed this. In the raw phenotype table, some of the samples are 'female'. Phenotypes were changed to be all male.	Partly. No batch info	age
GSE117859	GEO	53	608	608	49 ± 7.6	25 - 75	450K	100.0	Smoking + HIV		N/A	Yes	age + smoking
GSE117860	GEO	54	529	529	48 ± 7.8	25 - 75	450K	100.0	Smoking + HIV		N/A	Yes. More than half the CpGs were removed during pre-processing due to NA probes. After checking the probes this is a 450K dataset and not 850K as stipulated on GEO.	age + smoking
GSE147740	GEO	55	1129	1029	42 ± 7.8	26 - 59	EPIC	60.3	Normal		Yes. 3 Samples with incorrect sex were removed.	Yes. Removed samples without actual ages (samples with ages as a range)	age + sex
GSE152026	GEO	56	934	927	35 ± 13	18 - 64	EPIC	54.9	Psychosis / control		Yes. 1 Sample with incorrect sex removed	Yes	age + sex + disease status
GSE132203	GEO	57	795	795	42 ± 12	18 - 76	EPIC	28.2	Psychiatric disorders		Yes	Yes	age + sex + ethnicity + child abuse
GSE100264	GEO	58	386	386	50 ± 7.3	25 - 75	450K	100.0	Drug use + Hepatitis C + HIV+ / control		Yes	Yes	age + hepatitis C infection
GSE107080	GEO	59	405	405	48 ± 8.0	25 - 75	EPIC	100.0	Drug use + Hepatitis C + HIV+ / control		Yes	Yes	age + hepatitis C infection + race + smoking + artadherence
GSE116339	GEO	60	679	676	54 ± 13	23 - 88.5	EPIC	41.3	Polybrominated biphenyl exposure		Yes. 3 samples with incorrect sex removed	Yes	age + sex + PBB exposure
GSE168739	GEO	61	407	402	42 ± 10	19 - 61	EPIC	44.9	COVID-19		Yes. 5 samples with incorrect sex removed	Yes	age + sex
GSE197674	GEO	62	2138	2138	33 ± 10	6 - 66.4	EPIC	47.1	Survivors of childhood cancer		Yes. All samples clustered with the correct sex	Partly. Used their pre-processed matrix but filtered additional champ probes and pidsley cross-reactive probes. Performed combat for slide and array.	age + sex + abdominal pelvic rt + brain rt + chest rt + alkylating agent + anthracyclines + corticosteroids + epipodophyllotoxins + platinum + vincristine
GSE197676	GEO	63	282	282	36 ± 10	18.6 - 70.2	EPIC	45.6	Healthy / non-diseased		Yes	Yes	age + sex
			**36954**	**32136**									

GOLDN, Genetics of Lipid-Lowering Drugs and Diet Network Study; WHI, Women's Health Initiative; NAS, Normative Ageing Study; BIOS, Biobank-based integrative omics study; JHS, Jackson Heart Study; FHS, Framingham Heart Study; SATSA, Swedish Adoption/Twin Study of Ageing; GEO, Gene Expression Omnibus; dbGaP, Database of Genotypes and Phenotypes; N, Sample number; SD, Standard deviation; HIV, human immunodeficiency virus; COVID-19, coronavirus disease of 2019; QC, quality control

Table S2. Shannon entropy results in blood.

Dataset	Tissue	N	Age (mean ± SD)	Array	% Male	Entropy *p*-value	Effect	Stderr	Entropy *p*-value CTC	Effect CTC	Stderr CTC	ALL *p*-value	ALL effect	ALL stderr	NON *p*-value	NON effect	NON stderr
BIOS	Blood	1408	37.5 ± 13.9	450K	34.2	5.62461E-12	0.000108893	1.56711E-05	1.66797E-15	0.000109871	1.36385E-05	2.48E-28	0.000230373	2.04E-05	1.64E-05	-5.70E-05	1.64E-05
FHS	Blood	2498	66.3 ± 8.9	450K	45.8	0.1877697	3.86966E-05	2.93697E-05	2.78042E-05	9.45327E-05	2.25156E-05	0.036621	8.69E-05	4.16E-05	0.2459571	-2.85E-05	2.46E-05
GOLDN	Blood	967	48.9 ± 16.4	450K	47.4	5.824E-08	0.000245031	4.48189E-05	0.00002118	0.000164522	3.85012E-05	6.11E-15	0.000404495	5.10E-05	0.5808556	2.84E-05	5.14E-05
GSE100264	Blood	386	49.5 ± 7.3	450K	100	0.9039949	-1.177589e-05	9.756643e-05	0.7386943	-3.318863e-05	9.941942e-05	0.218594	0.000175854	0.000143	0.08593418	-0.000211998	0.000123135
GSE106648	Blood	279	41.2 ± 11.2	450K	27.6	0.000546446	0.000165539	4.73E-05	5.87E-08	0.000190881	3.42E-05	0.00012	0.000283737	7.27E-05	0.8445477	6.92E-06	3.52E-05
GSE107080	Blood	405	47.9 ± 8.0	EPIC	100	8.585942e-05	7.605117e-05	8.585942e-05	0.1803641	8.5567e-05	6.376264e-05	0.209893	0.000158405	0.000126	0.6157777	-3.81E-05	7.58E-05
GSE111629	Blood	564	69.3 ± 11.3	450K	56.2	0.09432215	7.60E-05	4.54E-05	0.02716773	7.68E-05	3.47E-05	0.023896	0.00014016	6.19E-05	0.8094899	-1.17E-05	4.87E-05
GSE115278 450K	Blood	355	46.9 ± 15.4	450K	35.8	0.5078759	3.8748E-05	0.000058459	0.4362365	4.20312E-05	4.20312E-05	0.264123	7.82E-05	6.99E-05	0.8314082	-1.49E-05	6.99E-05
GSE115278 EPIC	Blood	106	46.1 ± 9.6	EPIC	36.8	0.5765098	-8.57169E-05	0.000152996	0.9879491	-1.93584E-06	0.000127859	0.906673	-2.30E-05	0.000196	0.3511263	-0.000163515	0.000174581
GSE116339	Blood	676	53.9 ± 12.9	EPIC	41.3	0.5539667	1.750642e-05	2.956551e-05	2.050586e-05	6.457464e-05	2.050586e-05	0.731308	1.56E-05	4.55E-05	0.4511984	1.99E-05	2.64E-05
GSE117859	Blood	608	49.4 ± 7.6	450K	100	0.6654736	3.19082E-05	7.37624E-05	0.5534623	3.25011E-05	5.48163E-05	0.665474	3.19E-05	7.38E-05	0.02726577	-0.000142777	6.45E-05
GSE117860	Blood	529	48.1 ± 7.8	450K	100	0.1489717	9.180556e-05	6.352066e-05	0.07426908	8.187646e-05	4.57793e-05	0.095856	0.000163884	9.82E-05	0.8964397	-6.76E-06	5.19E-05
GSE120307	Blood	34	35.5 ± 11.0	450K	53	0.9649455	9.95E-06	0.000224609	0.897244	-2.31E-05	0.000177596	0.892103	3.69E-05	0.00027	0.9041536	-2.65E-05	0.000218004
GSE128235	Blood	532	47.7 ± 13.4	450K	42.7	0.7846098	8.58E-06	3.14E-05	0.00234564	9.61E-05	3.14E-05	6.86E-05	0.000175678	4.38E-05	0.2050711	-5.50E-05	4.33E-05
GSE132203	Blood	795	42.3 ± 12.3	EPIC	28.2	8.25274E-08	0.000229697	4.24405E-05	7.7381E-10	0.000221362	3.55529E-05	3.12E-10	0.000355602	5.58E-05	0.162107	6.88E-05	4.92E-05
GSE147740	Blood	1029	41.6 ± 7.8	EPIC	60.3	0.8578721	-1.34189E-05	7.49119E-05	0.4528796	4.86315E-05	6.47635E-05	0.485971	5.40E-05	7.74E-05	0.3093811	-9.26E-05	9.11E-05
GSE151278	Blood	70	47.1 ± 14.6	450K	61.4	0.05822633	0.0001772981	9.203258e-05	0.08005927	0.0001357616	7.640418e-05	0.021347	0.000247961	0.000105	0.4872273	8.21E-05	0.00011757
GSE152026	Blood	927	35.2 ± 12.8	EPIC	54.9	0.0006154573	0.0001244897	3.622545e-05	5.892645e-05	0.0001302386	3.227122e-05	2.08E-11	0.000294393	4.34E-05	0.06430396	-9.20E-05	4.96E-05
GSE157131 450K	Blood	272	66.7 ± 6.5	450K	100	0.1403107	0.0002125616	0.0001437215	0.5701558	7.182903e-05	0.0001263443	0.029841	0.000407517	0.000187	0.7411363	-5.07E-05	0.000153269
GSE157131 EPIC	Blood	946	62.6 ± 9.7	EPIC	100	0.0003969457	0.0002206612	6.206786e-05	0.002077936	0.000146279	4.74E-05	6.55E-07	0.000421581	8.42E-05	0.7718038	-1.75E-05	6.02E-05
GSE164056	Blood	143	25.8 ± 6.4	EPIC	34.3	0.00011632	0.000280024	0.00011632	0.01755237	0.000225162	9.36921E-05	0.001561	0.000495444	0.000154	0.891697	1.60E-05	0.000117325
GSE166611	Blood	32	38.5 ± 12.4	450K	0	0.3533241	0.0001497593	0.0001588485	0.6865235	5.329313e-05	0.000130776	0.104708	0.000371036	0.000222	0.1738786	-0.000147505	0.000105896
GSE168739	Blood	402	42.0 ± 10.3	EPIC	44.9	0.005971567	0.000194046	7.02016E-05	0.000830029	0.00015494	4.60011E-05	0.00116	0.000368954	0.000113	0.7208878	-2.16E-05	6.03E-05
GSE19711	Blood	262	64.9 ± 6.7	27K	0	0.3314212	0.000113503	0.000116644	0.7376015	2.90499E-05	8.66153E-05	0.180264	0.000204286	0.000152	0.8299482	2.31E-05	0.000107638
GSE197674	Blood	2138	32.9 ± 10.0	EPIC	47.1	4.40E-28	0.000319244	2.86E-05	1.72E-44	0.000335183	2.34E-05	3.18E-43	0.000513533	3.64E-05	0.000502173	8.19E-05	2.35E-05
GSE197676	Blood	282	35.8 ± 10.2	EPIC	45.6	7.01873E-05	0.000376987	7.01873E-05	4.1106E-09	0.000379745	6.25442E-05	8.73E-05	0.000635775	8.73E-05	0.4128983	5.81E-05	7.08E-05
GSE20236	Blood	93	32.0 ± 18.78	27K	0	0.2295936	5.89216E-06	4.87146E-06	0.03455883	8.24421E-06	3.8422E-06	0.094658	8.24E-05	4.88E-05	0.1785174	-6.90E-05	5.09E-05
GSE32148	Blood	44	19.7 ± 14.6	450K	47.9	0.558993	6.96E-05	0.000118187	0.03244751	0.000201963	9.13E-05	0.663959	6.74E-05	0.000154	0.5142416	7.29E-05	0.00011083
GSE40279	Blood	656	64.0 ± 14.7	450K	48.5	0.3204021	5.10554E-05	5.13436E-05	0.9272555	4.24542E-06	4.64825E-05	0.092281	9.91E-05	5.88E-05	0.5737251	-3.08E-05	5.47E-05
GSE41037	Blood	703	37 ± 16	27K	61.9	0.1592406	4.56E-05	3.23E-05	0.008152934	6.73E-05	2.53E-05	0.000591	0.000120773	3.50E-05	0.5985051	-1.81E-05	3.43E-05
GSE41169	Blood	93	31.6 ± 10.4	450K	29.5	0.2869219	0.000134718	0.000125766	0.06228045	0.000220851	0.000117007	0.103579	0.000233607	0.000142	0.9900006	1.85E-06	0.000147594
GSE42861	Blood	689	51.9 ± 11.8	450K	28.6	0.001366545	0.000159234	4.95317E-05	0.04421635	8.12344E-05	4.03001E-05	2.06E-06	0.000307344	6.42E-05	0.3254957	-4.59E-05	4.66E-05
GSE49904	Blood	71	55 ± 14.5	27K	31	0.2402566	0.000163367	0.000137914	0.8946044	1.60E-05	0.000120196	0.05103	0.000279712	0.000141	0.6982522	6.45E-05	0.000165601
GSE50660	Blood	463	55.4 ± 6.6	450K	70.5	0.6890406	-4.47802E-05	0.000111836	0.9971298	3.68951E-07	0.000102507	0.947926	7.25E-06	0.000111	0.3793442	-0.000114795	0.000130456
GSE51032	Blood	378	53.2 ± 7.2	450K	20.4	0.001526438	0.000451577	0.000141488	0.001150322	0.000400854	0.000122414	0.000101	0.000600497	0.000153	0.1682637	0.000215158	0.000155877
GSE53740	Blood	162	68.1 ± 10.3	450K	47.9	0.1677049	0.000164121	0.000118422	0.08266189	0.000105605	6.05E-05	0.156757	0.000247893	0.000174	0.5929959	4.94E-05	9.22E-05

GSE53840	Blood	109	52 ± 8	450K	100	0.2500448	0.000164896	0.00014258	0.1407098	0.000117615	7.92472E-05	0.429937	0.000180077	0.000227	0.19465	0.000145058	0.000111145
GSE55763	Blood	2639	51 ± 10.1	450K	67.7	3.15012E-24	0.000107633	1.04972E-05	3.25478E-35	0.000104553	8.32351E-06	4.77E-36	0.000238909	1.88E-05	2.13E-07	-7.01E-05	1.35E-05
GSE58045	Blood	172	57.2 ± 8.2	27K	0	0.001603156	0.00078535	0.000244892	0.9427179	-1.47415E-05	-1.47415E-05	7.35E-05	0.001002084	0.000247	0.05238299	0.000568455	0.00029097
GSE67705	Blood	186	46.1 ± 8.8	450K	100	0.00380295	8.38585E-05	2.86073E-05	0.001462808	0.000156706	2.61127E-05	0.000166	0.000143254	3.73E-05	0.9221143	4.15E-06	4.24E-05
GSE67751	Blood	92	49.5 ± 8.9	450K	45.7	0.6995618	8.66E-05	0.00022372	0.4221127	-0.000141879	0.000175932	0.209037	0.000333099	0.000263	0.246199	-0.000244177	0.000209193
GSE69138	Blood	184	74.8 ± 12.6	450K	51.1	0.8784567	-1.70775E-05	0.000111515	0.9707839	3.05626E-06	8.33324E-05	0.684125	-6.07E-05	0.000149	0.7793944	3.06E-05	0.000109034
GSE72774	Blood	507	69.6 ± 11.2	450K	55.4	0.2455719	9.69E-05	8.34E-05	0.02605122	0.0001603	7.18E-05	0.085205	0.00015757	9.14E-05	0.8923563	1.39E-05	0.000102413
GSE72775	Blood	335	70.2 ± 10.3	450K	58.8	0.09070863	0.000187568	0.000110556	0.00390439	0.000246771	8.49139E-05	0.009118	0.000323684	0.000123	0.9918559	1.40E-06	0.000137433
GSE72776	Blood	84	66.4 ± 11.7	450K	59.5	0.2467337	0.000216987	0.000185992	0.6720531	5.85E-05	0.00013777	0.010186	0.000572139	0.000218	0.2100965	-0.000261696	0.000207169
GSE134429	Blood	63	56.0 ± 12.7	EPIC	11.1	0.01163675	0.000651829	0.00025701	0.3016693	0.00022742	0.000219863	3.67E-07	0.001083024	0.000209	0.7060233	0.000135957	0.000360143
GSE77445	Blood	85	33.8 ± 15.9	450K	50.5	0.2884682	-0.000124334	0.000116111	0.3170136	-0.000104965	0.00010404	0.351017	-9.59E-05	1.02E-04	0.2711624	-0.00015937	1.44E-04
GSE80417	Blood	636	40.4 ± 15.0	450K	59.3	0.1578088	4.11E-05	2.91E-05	0.005250155	7.60E-05	2.71E-05	0.001604	9.04E-05	2.85E-05	0.4897902	-2.51E-05	3.64E-05
GSE85311	Blood	38	47.4 ± 17.9	450K	71	0.9905637	2.223144e-06	0.0001866718	0.549951	-9.716753e-05	0.000161003	0.06426	0.000306489	0.000161	0.1166512	-0.000407661	0.000253578
GSE87571	Blood	728	47.4 ± 21.0	450K	46.7	3.21171E-33	0.000260116	2.05873E-05	6.84639E-19	0.000150763	1.65182E-05	5.43E-42	0.000457339	3.16E-05	0.4415698	-1.27E-05	1.65E-05
GSE96879	Blood	90	51.6 ± 14.4	450K	0	0.01628075	0.0001682668	6.869033e-05	0.007405148	0.0001286493	4.692491e-05	0.038121	0.000228333	0.000108	0.1831673	8.75E-05	6.52E-05
GSE99624	Blood	48	67.2 ± 9.9	450K	18.7	0.2631376	-0.000161301	0.000142382	0.5391254	-6.37106E-05	0.000102965	0.723378	-7.80E-05	0.000219	0.00013508	-0.000116006	0.00013508
JHS	Blood	1747	55.7 ± 12.3	EPIC	37	0.00319957	8.835696e-05	2.993141e-05	4.047926e-05	9.463708e-05	2.299669e-05	4.56E-05	1.53E-04	3.73E-05	2.13E-07	-7.01E-05	1.35E-05
NAS	Blood	1454	74.5 ± 7	450K	100	0.4407512	-4.77873E-05	6.19699E-05	0.2034583	7.78616E-05	6.11959E-05	0.049396	0.00015854	8.06E-05	0.5675632	-3.34E-05	5.84E-05
SATSA	Blood	1071	73.1 ± 9.7	450K	39.7	0.8347775	1.59187E-05	7.62863E-05	0.08817062	0.000113456	6.64096E-05	0.138618	-0.000112596	7.60E-05	0.003085382	0.000243825	8.22E-05
WHI	Blood	1871	64.2 ± 7.0	450K	0	0.3131826	4.41529E-05	4.3766E-05	0.04760497	6.50322E-05	3.28084E-05	0.27251	6.98E-05	6.36E-05	0.8377477	7.85E-06	3.83E-05

GOLDN, Genetics of Lipid-Lowering Drugs and Diet Network Study; WHI, Women's Health Initiative; NAS, Normative Ageing Study; BIOS, Biobank-based integrative omics study; JHS, Jackson Heart Study; FHS, Framingham Heart Study; SATSA, Swedish Adoption/Twin Study of Ageing; N, Sample number; SD, Standard deviation; Stderr, Standard error; CTC, cell-type corrected; ALL, All age-associated CpGs; NON, non-age-associated CpGs

Table S3. Entropy results for three categories of age-associated CpGs.

A

	DMPs only			DMPs only CTC		
	p-value	effect	stderr	p-value	effect	stderr
BIOS	**0.00263494**	0.000046	1.53E-05	**0.001221366**	0.000046509	1.44E-05
GSE87571	**2.13E-18**	0.000176	1.96E-05	**2.85E-10**	0.000111197	1.74E-05

B

	DMP/ VMP			DMP / VMP CTC		
	p-value	effect	stderr	p-value	effect	stderr
BIOS	**1.29E-41**	0.000383	2.75E-05	**5.30E-78**	0.0003835	1.92E-05
GSE87571	**1.88E-47**	0.000649	4.16E-05	**2.81E-32**	0.000352149	2.83E-05

C

	VMPs only			VMPs only CTC		
	p-value	effect	stderr	p-value	effect	stderr
BIOS	**1.18E-12**	-0.0000900	1.25E-05	**3.84E-11**	0.00030754	3.08E-04
GSE87571	0.02844887	-0.0000415	1.89E-05	0.03538065	-0.00003671	1.74E-05

BIOS, Biobank-based integrative omics study; DMPs, differentially methylated positions; VMPs, variably methylated positions; stderr, standard error; CTC, cell type correction.

Table S4. Entropy results in datasets containing isolated cell types.

Dataset	Ref	Cell type	N	Age ± sd	p-value	effect	stderr	ALL p-value	ALL effect	ALL stderr	NON p-value	NON effect	NON stderr
GSE56046	79	Monocytes	1201	60 ± 9.5	0.2551	0.000017	0.000015	0.572209	0.572198	0.000020	**0.000917**	0.000048	0.000014
GSE59065	80	CD4+ T cells	100	52 ± 24	**1.22E-07**	0.000321	0.000056	**2.49E-11**	0.000518	0.000069	0.506980	0.000045	0.000067
GSE59065	80	CD8+ T cells	100	53 ± 24	**2.01E-11**	0.000556	0.000073	**6.37E-13**	0.000811	0.000098	**0.003355**	0.000199	0.000066
GSE56581	81	CD4+ T cells	211	59 ± 8.7	0.2107	0.000065	0.000052	0.083408	0.000137	0.000079	0.254963	0.000031	0.000027
GSE137593	82	CD4+ T cells	101	56 ± 14	0.3264	-0.00012	0.000123	0.901693	-0.00002	0.000146	0.111324	0.000255	0.000159
GSE137594	83	B cells	119	56 ± 14	0.7809	-0.00003	0.000104	0.702303	-0.00005	0.000135	0.997297	0.000000	0.000100

N, Sample number; SD, Standard deviation; Stderr, Standard error; CTC, cell-type corrected; ALL, All age-associated CpGs; NON, non-age-associated CpG
