# Supplementary material for: A comprehensive map of the aging blood methylome in humans
Source: Genome Biol. 2024 Sep 6;25:240. doi: 10.1186/s13059-024-03381-w (PMC11378482; doi:10.1186/s13059-024-03381-w)
Supplement: Supplementary file 3 — Additional file 3: The peer review history. [file 13059_2024_3381_MOESM3_ESM.docx]

**Review history**

**First round of review**

**Reviewer 1**

In this work, the authors perform a large analysis of changes in the blood methylome with age. They find widespread changes in differentially methylated positions and in increased divergence with age, plus other analyses. The manuscript is clear and well written, and I think will make an important contribution to the literature. I only have a few minor concerns and suggestions, as detailed below.

One of the major findings from this study is that nearly half of the tested methylation sites change with age. This is a very large effect, and I wonder how this compares to other cases. For instance, how many differentially methylated positions would you find between, say, two different cell types?

Line 50: I don't think it's correct to say that ageing is governed by the hallmarks, these are merely hypothesis that by and large are not supported by empirical evidence. For a recent paper on this please see:
https://www.nature.com/articles/s41588-023-01627-0

Line 177 and others: strange symbol before test.

Lines 350-353: an age-related loss of cell identity has also been observed in aged human tissues based on gene expression data:
https://pubmed.ncbi.nlm.nih.gov/37884865/

I think such cellular changes could reflect the increase in entropy observed by the authors, namely a drift towards a methylation fraction of 50%.

Lastly I found the figures very difficult to read, figure 1 was just a blur.

Overall, I think this is a very interesting work that will make a valuable contribution to the literature, though a few aspects could be improved.

It is possible that some of my comments reflect misunderstandings of mine. If so then I would suggest that the authors use my misunderstandings as an indication that such points might be made clearer in the manuscript.

It my usual policy to reveal my identity to the authors: Joao Pedro de Magalhaes.

**Reviewer 2**

Seale et. al performed a comprehensive analysis of over 32,000 blood methylomes revealed a significant proportion of differentially and variably methylated sites with age, with many overlapping, and showed that the increase in entropy is primarily driven by DMPs shifting towards a 50% methylation fraction, despite some DMPs having anti-entropic effects, with VMPs and entropy measurements being moderately sensitive to cell type composition changes. However, some concerns should be further addressed.

1. The innovation of the study can be considered limited if it only focuses on the overlap between VMPs and DMPs and the distribution of these probes, as the research team has discussed this phenomenon in a previous review.
2. In Figure 5, the color of the heatmap and the dimensions of the blocks depicted should be clearly defined within the Figure legend to convey their specific meanings.
3. The authors' findings report more than 300,000 differentially methylated sites (DMPs), raising a question about the utilization of 56 databases from different sequencing platforms. If this calculation is based on data from the 850K platform, what is the role of the 27K and 450K chip data in this study.
4. The authors have identified an unusually high number of differentially methylated sites. It may be prudent to consider whether the screening criteria, like the threshold for changes in methylation levels per year of age, should be raised appropriately to ensure the accuracy and relevance of the results.
5. The following minor points should be checked:
a. L177, L204, L279, L281: test p-value and other similar typos.
b. L187: Should this sentence be merged into the above paragraph?
c. L818: "(top panel)" should be "(first panel)".
d. There is no correlation coefficient and P-value on plots in Figure 2-4. The correlation method should be mentioned on the figure legends.
e. L859: Please check if (A), (B) and (C) in this line is corresponding to the Figure.
f. L860: "antientropic" should be "anti-entropic".
g. L863: Please correct "2 tests".

**Reviewer 3**

This manuscript is of interest and the methods used look appropriate and sensible. I think the meta-analysis approach used for all 3 parts of the analysis is correct and the interpretation of the findings is a worthwhile advance. The text mentions multiple versions of the Illumina platform, which is important because they are very different in number of features. Ideally a version of supplementary table 1, which does a good job of presenting the cohorts, should be in the main manuscript and the authors should provide more detail on how they deal with the fact that they have differing amounts of data on subsets of the features.

My ability to properly assess the manuscript was severely compromised by problems that are only partly the authors' fault and convey to me a profound lack of respect for the reviewers and the review process by the journal. To me it's unacceptable that the code used in the analysis is not available for review. Worse, I have not been able to review the figures, as they are presented in the reviewer PDF in unusable form. I am sure that authors' original version of the figures was worth looking at but after processing there is literally nothing recognisable in figure 1. Figure 2 onwards are such low resolution bitmaps that the labelling is unreadable. What are the editorial staff thinking when they give reviewers this to look at? What's the point of even sending the paper out for review if you tie the reviewers hands behind their back? In this case I am fairly confident that the authors have done reasonable work but I am taking a lot on faith and I've not scrutinised the figures at all.

**Authors’ response to reviewers**

Reviewer #1:
In this work, the authors perform a large analysis of changes in the blood methylome with age. They find widespread changes in differentially methylated positions and in increased divergence with age, plus other analyses. The manuscript is clear and well written, and I think will make an important contribution to the literature. I only have a few minor concerns and suggestions, as detailed below.

1. One of the major findings from this study is that nearly half of the tested methylation sites change with age. This is a very large effect, and I wonder how this compares to other cases. For instance, how many differentially methylated positions would you find between, say, two different cell types?

Response:
We see a very large “effect” of age on the epigenome in terms of the number of CpGs that change with age, and the reproducibility of the effect across several datasets. This was detectable due to the high degree of statistical power that we generated from our sample size (>32,000), the distribution of samples across a very broad age range, and using a meta-analysis study design. This gives us confidence that the change with age we observed is more likely a probable scenario, than can be expected by chance.
With that said, we generally refer to effect size as the magnitude of the change, and not the number of features. If we look at the cell-type specific DNA methylation differences, they are of a much larger effect size (i.e. the magnitude of change) than those associated with ageing. For example, between cell types the number of CpGs that display large differences (>50%) are in the 10s of 1000s. However, with ageing, it is rare to find CpGs that display more than 30% DNA methylation difference during a whole lifetime. In fact, the overwhelming majority of age-related DMPs only display 5 – 10% DNA methylation difference over a ~ 50 year period. Therefore, while cell-type specific DMPs have a larger effect size than age DMPs, age-DMPs may be more numerous, which makes biological sense, as we see a gradual erosion of the methylome from the ground state, and this is a genome-wide effect.

2. Line 50: I don't think it's correct to say that ageing is governed by the hallmarks, these are merely hypothesis that by and large are not supported by empirical evidence. For a recent paper on this please see:
https://www.nature.com/articles/s41588-023-01627-0

Response:
Thank you for sharing this insightful paper. We have revised the manuscript to now read:
Lines 48 – 50: “Ageing is initiated at the basic level of biological organisation and is hypothesised to be underpinned by 12 interconnected hallmarks [2], including alterations to the epigenome; a focus of this research [2].”

3. Line 177 and others: strange symbol before test.
Response:
Thank you for identifying this error. It may be an error with the manuscript upload.

4. Lines 350-353: an age-related loss of cell identity has also been observed in aged human tissues based on gene expression data:
https://pubmed.ncbi.nlm.nih.gov/37884865/
Response:
Thank you for sharing this paper. We have now added this manuscript to the references (34), and included the following in the manuscript:

Lines 354 – 355: “This phenomenon has also been observed in human tissues based on gene expression data, whereby 40% of the tissues lose their cellular identity during ageing [34].”

5. I think such cellular changes could reflect the increase in entropy observed by the authors, namely a drift towards a methylation fraction of 50%.
Response: Thank you for your comment. We have added additional rationale for interpreting the influence of cell type changes to entropy:

Lines 304 - 307: “These results suggest that changes in cell type composition during ageing partially account for age-related increases in entropy. Moreover, the measure of entropy that we have used may be extremely sensitive to confounders that induce relatively small shifts in the global DNA methylation distribution.”

6. Lastly I found the figures very difficult to read, figure 1 was just a blur.
Response:
Apologies, there may have been an error with the figure upload. We have now uploaded a high-resolution figure.
Overall, I think this is a very interesting work that will make a valuable contribution to the literature, though a few aspects could be improved.
Response:
We thank the reviewer for their positive comments.


Reviewer #2:
Seale et. al performed a comprehensive analysis of over 32,000 blood methylomes revealed a significant proportion of differentially and variably methylated sites with age, with many overlapping, and showed that the increase in entropy is primarily driven by DMPs shifting towards a 50% methylation fraction, despite some DMPs having anti-entropic effects, with VMPs and entropy measurements being moderately sensitive to cell type composition changes. However, some concerns should be further addressed.

1. The innovation of the study can be considered limited if it only focuses on the overlap between VMPs and DMPs and the distribution of these probes, as the research team has discussed this phenomenon in a previous review.

Response: Thank you for your comment. Indeed, this phenomenon was discussed as a hypothesis in our recent review paper, however, such a large-scale EWAS study investigating the differences in DMPs and VMPs had never been carried out. Moreover, the novelty lies in the contribution of these features of ageing to entropy. Here, we investigated DMPs and VMPs in a much larger cohort (~10x larger), and following a more robust statistical approach, we found that the overlap between phenomena was much larger than originally hypothesised in the review.

2. In Figure 5, the color of the heatmap and the dimensions of the blocks depicted should be clearly defined within the Figure legend to convey their specific meanings.
Response: Thank you for identifying this error. We have amended the figure legend to read as follows:
Lines 908 - 914: “…C) Distribution of entropic (blue) and anti-entropic differentially methylated positions (DMPs) (red) and non-DMPs (grey) in chromatin states from peripheral blood mononuclear cells (PBMCs); The grids under the graph represent the residuals from the ꭓ2 tests, with the size of the blocks in the grid being proportional to the cell’s contribution. Sky blue indicates over-representation or enrichment in the chromatin state, and navy represents under-representation or depletion in the chromatin state.”

3. The authors' findings report more than 300,000 differentially methylated sites (DMPs), raising a question about the utilization of 56 databases from different sequencing platforms. If this calculation is based on data from the 850K platform, what is the role of the 27K and 450K chip data in this study.

Response: CpG sites included in the 850K platform encompass all those from both the 450K and 27K arrays, ensuring conservation across platforms. During preprocessing, some CpG sites may fail quality control and are subsequently discarded. Despite not achieving a perfect overlap of CpG sites across each dataset, each CpG site is meta-analysed to maximise inclusion across datasets. This approach aims to optimise sample size for each meta-analysis, thereby enhancing statistical power and robustness of the findings. We have included the following in the paper:
Lines 412-416: “Moreover, we utilised multiple versions of the Illumina arrays to ensure maximise the inclusion of CpGs across the various datasets. CpGs in the 850K encompass all those from both the 450K and the 27K platforms, ensuring conservation across platforms. Despite not achieving a perfect overlap across datasets, each CpG is individually meta-analysed, and this approach optimises the sample size for each meta-analysis.”

4. The authors have identified an unusually high number of differentially methylated sites. It may be prudent to consider whether the screening criteria, like the threshold for changes in methylation levels per year of age, should be raised appropriately to ensure the accuracy and relevance of the results.

Response: Our approach focused on establishing significance through consistency across at least three individual datasets, each with specific age ranges and sample sizes, rather than emphasizing the magnitude of methylation changes per year of age. Additionally, we applied a stringent false discovery rate (FDR) threshold of <0.005. Our rationale behind this method was to prioritise associations that were consistently observed across multiple datasets, regardless of the size of the effect. Given the current understanding, we lack a clear criterion for determining what constitutes a meaningful functional effect size in this context. We too were surprised by the number of DMPs identified, however, this highlights the sheer magnitude and omnipresence of age-related changes in the methylome.

5. The following minor points should be checked:
a. L177, L204, L279, L281: test p-value and other similar typos.
Response: This may be an error with the upload from the journal website as there is no error in the manuscript.

b. L187: Should this sentence be merged into the above paragraph?
Response: Thank you, this has been amended in text and the sentence is now part of the paragraph above.

c. L818: "(top panel)" should be "(first panel)".
Response: Corrected.
Lines 857: “… (first panel”)”

d. There is no correlation coefficient and P-value on plots in Figure 2-4. The correlation method should be mentioned on the figure legends.
Response: Thank you for your feedback. These graphs are not correlation plots, they are methylation plots that simply serve to highlight the reproducibility of the linear change of age at a single CpG site across three independent datasets. The significance of the association is also not represented by a single dataset, but rather the meta-analysis FDR.
We have made the following amendment to the figure legends:
Figure 2B: “…A forest plot of a highly significant CpG (cg16867657), FDR < 2.2 x 10-16. The dataset and sample size are on the left side of the plot, with the corresponding effect size and errors represented by the point and error bars. The meta-analysis effect size is represented by the purple polygon (0.45% change per year of age).”
Figure 3B: “…A forest plot of the top VMP (cg21899500), FDR < 2.2 x 10-16, that is hypermethylated with age and increases in variance with age. The dataset and sample size are on the left side of the plot, with the corresponding effect size and errors represented by the point and error bars. The methylation plots for this CpG from three independent blood cohorts, including GSE40279, GSE152026 and Jackson Heart Study (JHS) are displayed on the far right, with age on the x-axis and methylation fraction (MF) as a percentage on the y-axis. Each point on the plot represents a single sample. B) Venn diagram of the overlap between DMPs and VMPs. The numbers in each circle represent the number of CpGs that are DMPs only (left), both DMPs and VMPs (middle), and VMPs only (right). The arrows point to an example of each category of age-related CpG displayed as a methylation plot, with age on the x-axis and methylation fraction (MF) as a percentage on the y-axis. Each point represents a single sample.”
Figure 4: “…The meta-analyses effect sizes are represented by the orange and light green polygons. On the x-axes is the change in entropy per decade of age (left: -0.0003 change in entropy per 10 years of age p-value <0.0001; right: 0.002 per decade of age (p-value <0.0001), and on the y-axes are the effect sizes and standard error measurements from the independent epigenome-wide association study (EWAS).”
e. L859: Please check if (A), (B) and (C) in this line is corresponding to the Figure.
Response: Thank you for identifying this error. The figure legend has been corrected as follows:
Lines 901 - 914: “Figure 5. Contribution of differential methylation to changes in entropy with age. A) A pie chart of the proportion of DMPs that are entropic and converge to the mean (blue) and the proportion of DMPs that are anti-entropic and diverge from the mean (red) for the BIOS dataset in blood. The arrows point to hypothetical graphs that illustrate the aggregate regression lines for the CpGs that converge (left) and diverge (right) with age. B) In the far left (blue) and far right (red) panels are the methylation plots for two CpG sites, respectively, that are highly or lowly methylated in young (blue) and converge to the mean with age, and two methylation plots that are intermediately methylated in young (red) and diverge from the mean with age. C) Distribution of entropic (blue) and anti-entropic differentially methylated positions (DMPs) (red) and non-DMPs (grey) in chromatin states from peripheral blood mononuclear cells (PBMCs); The grids under the graph represent the residuals from the ꭓ2 tests, with the size of the blocks in the grid being proportional to the cell’s contribution. Sky blue indicates over-representation or enrichment in the chromatin state, and navy represents under-representation or depletion in the chromatin state.
f. L860: "antientropic" should be "anti-entropic".
Response: Thank you for identifying the error. This has been corrected in the Figure legend.
Lines 909: “…anti-entropic”
g. L863: Please correct "2 tests".
Response: There is no error in the manuscript, it is an error with the upload on the journal website.

Reviewer #3:
1. This manuscript is of interest and the methods used look appropriate and sensible. I think the meta-analysis approach used for all 3 parts of the analysis is correct and the interpretation of the findings is a worthwhile advance. The text mentions multiple versions of the Illumina platform, which is important because they are very different in number of features. Ideally a version of supplementary table 1, which does a good job of presenting the cohorts, should be in the main manuscript and the authors should provide more detail on how they deal with the fact that they have differing amounts of data on subsets of the features.

Response: Thank you for the suggestion. As Table 1 is very large (spans two horizontal A4 pages), we believe it is better suited to be in the supplementary material, which is easily accessible for the reader. Regarding your suggestion on providing more detail on the differing amounts of data on subsets of the features, we have included the following:
Lines 412 – 416: “Moreover, we utilised multiple versions of the Illumina arrays to ensure maximise the inclusion of CpGs across the various datasets. CpGs in the 850K encompass all those from both the 450K and the 27K platforms, ensuring conservation across platforms. Despite not achieving a perfect overlap across datasets, each CpG is individually meta-analysed, and this approaches optimises the sample size for each meta-analysis.”

My ability to properly assess the manuscript was severely compromised by problems that are only partly the authors' fault and convey to me a profound lack of respect for the reviewers and the review process by the journal. To me it's unacceptable that the code used in the analysis is not available for review. Worse, I have not been able to review the figures, as they are presented in the reviewer PDF in unusable form. I am sure that authors' original version of the figures was worth looking at but after processing there is literally nothing recognisable in figure 1. Figure 2 onwards are such low resolution bitmaps that the labelling is unreadable. What are the editorial staff thinking when they give reviewers this to look at? What's the point of even sending the paper out for review if you tie the reviewers hands behind their back? In this case I am fairly confident that the authors have done reasonable work but I am taking a lot on faith and I've not scrutinised the figures at all.

Response: The authors sincerely apologise that the figures were not of high quality after they were processed by the journal website. We assure you that they are of a high standard and the quality will not be compromised upon publication. The authors thank the reviewer for their honest feedback and hope that this is taken into consideration by the journal. The code is now available on github and Zenodo for your perusal.
